# An optimized modeling for in-situ stresses based on Rhino accurate modeling and large-scale transverse isotropic theory

**DOI:** 10.1038/s41598-023-28039-8

**Published:** 2023-01-13

**Authors:** Weihua Song, Huice Jiao, Xiaotian Xu, Ping He

**Affiliations:** 1 College of Mining, Liaoning Technical University, Fuxin, 123000 Liaoning China; 2 Ecological Restoration Technology Center, Shandong Province Research Institute of Coal Geology Planning and Exploration, Jinan, 250104 Shandong China; 3 Chief Engineer’s Office, The Fifth Coal Mine of Pingdingshan Tianan Coal Mining Co., LTD, Pingdingshan, 467000 Henan China

**Keywords:** Coal, Geophysics

## Abstract

In-situ stresses are significantly important for underground mining geotechnical design and coal seam gas management in underground coal mines. Aiming at regions with geological structures, this paper proposes an in-situ stress inversion method combining Rhino precise modeling and transverse isotropy theory, and tests it near the Guodishan fault. The results show that the application of Rhino precise modeling and transversely isotropic model in in-situ stress inversion shows advantages in improving the accuracy of in-situ stress calculation. In addition, based on the inversion analysis of in-situ stress near the Guodishan fault, we believe that the fault structure has a significant impact on the distribution of in-situ stress. Within the elevation range of − 400 m to − 800 m, the horizontal stress and vertical stress of the footwall are greater than those of the hanging wall. Moreover, the K ratio near the fault is generally less than 1, but the K ratio in the footwall is usually greater than that in the hanging wall, indicating that the tectonic stress has a stronger influence on the footwall. It is worth noting that the stress gradient near the fault is higher, which may lead to higher disaster risk.

## Introduction

The in-situ stress exists in the underground rock mass before operation and it is the cause of deformations and damages in the rock during the mining operations in either open pit or underground^1^. The distribution of the in-situ stresses is highly uneven due to the tectonic movement in earth. The conventional methods can only capture the in-situ stresses in the measurement points and hence are incapable of extrapolating the in-situ stresses in the large area. In addition, it is very hard to extrapolate the in-situ stresses in the whole area as the underground environment is too harsh for the measurement plus the measurement is normally super expensive. To date, two methods have been developed to determine the in-situ stresses in the region being in-situ stress estimation method and comprehensive analysis method of in-situ stress^2^. The in-situ stress estimation method, including Heim’s hypothesis, lateral pressure coefficient method, etc., is simple but its accuracy is compromised. By contrast, a typical comprehensive analysis method of in-situ stress, in-situ stress inversion method, has become more and more popular given its relatively higher accuracy.

Various in-situ stress inversion methods have been investigated by numerous researchers in recent years. Jin et al. captured the in-situ stresses distribution in Haihetan Hydraulic Power Station through inversion method along with the geological data, field measurement data and FLAC^3D^ numerical simulation results as well as Artificial Neural Network algorithms^3^. Zhang et al. used two phase optimisation method to investigate the in-situ stress field in Huangdeng Hydraulic Power Station in order to assess the rock stability in the underground openings^4^. Liu et al. developed an in-situ stress inversion approach based on Support Vector Regression (SVR) method and hence implemented in Coal Mine No. 1 owned by Pingdingshan Mining Group in China^5^. Considering the inconsistent mechanical properties of rock mass in different directions, Yu assigned transverse isotropic parameters to each rock layer and used multiple regression to predict the original in-situ stress^6^. Zhang established a numerical simulation research model considering the fault structure and surface characteristics, and used the neural network optimized by genetic algorithm to establish the nonlinear relationship between the measured and simulated values of variable boundary conditions, and then inverted the regional in-situ stress^7^. Xu et al. conducted in-situ stress field tests near fA42 thrust fault in Shihao Coal Mine, and then used multiple linear regression to retrieve regional stress field^8^. Zhu et al. established a 3D geological model in Rhino for Changcun Coal Mine and thereafter used FLAC^3D^ to extrapolate the in-situ stress in the rock mass^9^. Ning et al. used stepwise regression analysis and FLAC^3D^ numerical simulation method to invert and analyze the in-situ stress field of a valley in the upper reaches of Lancang River, obtained the spatial and temporal distribution characteristics of the in-situ stress field, and then discussed the influence of stress unloading effect on slope stability^10^. Zhou et al. proposed a piecewise single-hole inversion method using multiple regression model to characterize in-situ tunnel stress field on the basis of discussing the influence of tunnel longitudinal size effect on inversion accuracy, and applied it to the study of in-situ stress in southwest China^11^.

However, most of these methods are applicable to areas with simple geological structure and small stratigraphic dip. For areas with complex geological conditions and structures, there will be a large deviation between the numerical simulation results and the measured geostress. Such a deviation might be attributed to the error in the geological model development process or the oversimplification for the mechanical properties in the rocks, numerical model and numerical calculations. Therefore, the author introduces an inversion method of in-situ stress based on large-scale transversely isotropic model. That is, in the numerical simulation stage, the physical and mechanical properties of the small rock mass still use the isotropic model. However, in the stage of multiple linear regression, large-scale transverse isotropic model is used to conduct linear regression for horizontal and vertical in-situ stress respectively to improve the accuracy of in-situ stress inversion.

## Fundamentals

### In-situ stress regression analysis method

The main idea of the in-situ stress regression analysis method is to calculate the simulated values of in-situ stress under the independent action of each main factor affecting the in-situ stress based on the three-dimensional geological model of the study area, and establish the regression equation of the initial in-situ stress field with the measured values^12^.

**Figure 1:**
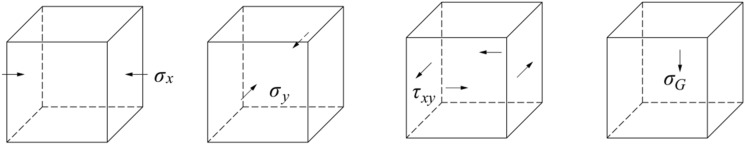
Decomposition of boundary stress.

The in-situ stress is affected by many factors such as topography, lithology, geological structure, ground temperature and groundwater, but the gravity and tectonic movement are the main factors for the formation of the in-situ stress^13^. Therefore, we can simplify the complex in-situ stress into self-weight stress and tectonic stress. At present, the hydraulic fracturing method is widely used in the in-situ stress testing, so the shear stress in the vertical plane cannot be measured. Therefore, we can decompose the ground stress on the block into four simple boundary stress forms, as shown in the Fig. 1.

Under the natural stress state of rock and soil, it can be considered that the rock and soil are in equilibrium without continuous plastic deformation change, and the in-situ stress of each point inside the plot can be considered as superposition of boundary stress. We take the actual in-situ stress as the dependent variable, and the results of four boundary stresses applied separately as the independent variable. Then, based on the linear superposition principle, we can obtain the regression Eq. (1) of the actual in-situ stress.

1 $$\sigma_{d} = b_{0} + b_{1} \sigma_{g} + b_{2} \sigma_{x} + b_{3} \sigma_{y} + b_{4} \tau_{xy} ,$$

where $\sigma_{d}$ is the actual in-situ stress, $\sigma_{g}$ is the stress caused by gravity,$\sigma_{x}$,$\sigma_{y}$ and $\tau_{xy}$ are the stress caused by horizontal x direction extrusion, horizontal y direction extrusion and shear in xy plane respectively; $b_{0} \sim b_{4}$ is the regression coefficient.

### Large-scale transversely isotropic model

In the previous inversion of in-situ stress^14–16^, rock mass is generally assumed to be homogeneous and isotropic medium. Although this assumption has played a positive role in understanding the mechanical characteristics of rock mass materials^17,18^, a large number of indoor and outdoor tests and engineering examples show that most rock masses, especially layered rock masses, are characterized by discontinuity and heterogeneity. Especially for giant blocks with length and thickness of several kilometers, rocks with different mechanical properties are stacked together in layers. As a result, the blocks show different properties in horizontal and vertical directions, which conform to the characteristics of transverse isotropy. If the whole block is still regarded as an isotropic elastic body, the inversion results of in-situ stress will be distorted.

For the inversion process of in-situ stress, if the transversely isotropic model is used in the numerical simulation stage, it is difficult to obtain the transversely isotropic mechanical parameters of rock masses in each layer due to the large scale of the block. At the same time, the thickness of each rock stratum is smaller than the thickness of the whole block, which will lead to large cumulative error.

Therefore, in the numerical simulation stage, the physical and mechanical properties of small rock mass still use the isotropic model. However, in the multiple linear regression stage, the large-scale transversely isotropic model is used to conduct linear regression for the horizontal and vertical in-situ stress respectively, so as to improve the accuracy of in-situ stress inversion.

### Precise 3D geological model by rhino

3D geological modeling is the process of establishing digital models of underground geological bodies based on various original data, and is the basis of geophysical research^19^. At present, there are many 3D geological modeling software used in different fields and specialties, and many scholars have also conducted in-depth research on 3D geological models. Ren et al. digitizes the known geological and geographic information into control points and line strings^20^. Then, high-quality three-dimensional volume models are constructed using SKUA Gocad software, and tetrahedral mesh is generated using Tetgen mesh generation program; Li et al. proposes an efficient 3D parametric modeling method based on NURBS for geological problems of engineering scale^21^; Zhang et al. used QuantyMine 3D mining software to build super large 3D geological models, scientifically expressed the unique metallogenic environment of the super large manganese ore field in Songtao County, Guizhou Province, and revealed the metallogenic mechanism and occurrence^22^.

Building an accurate 3D geological model is also an important means to improve the inversion accuracy. The coal and rock strata are undulating surfaces, and the general modeling software is not effective in building surfaces, nor can it accurately reflect the actual stratum extension. Therefore, it is very important to select a modeling software that can accurately establish the surface model for the accuracy of in-situ stress inversion. Rhino is a professional modeling software, which has been widely used in 3D animation, manufacturing, scientific research and other fields. More importantly, Rhino can automatically and quickly form accurate surfaces, and many numerical simulation software (such as FLAC^3D^, UDEC and 3DEC) are compatible, which can make up for the common shortcomings of the lack of model development ability in these numerical simulation software.

Based on a large number of borehole histograms in the coal mine area, the author formed the contour lines of F_16-17_ coal seam on both sides of the fault, and established an accurate F_16-17_ coal seam model using Rhino. Then other strata and ground are established in turn to form a more accurate three-dimensional geological model, which lays a good foundation for improving the accuracy of in-situ stress inversion.

## Geological model development

### Background

**Figure 2:**
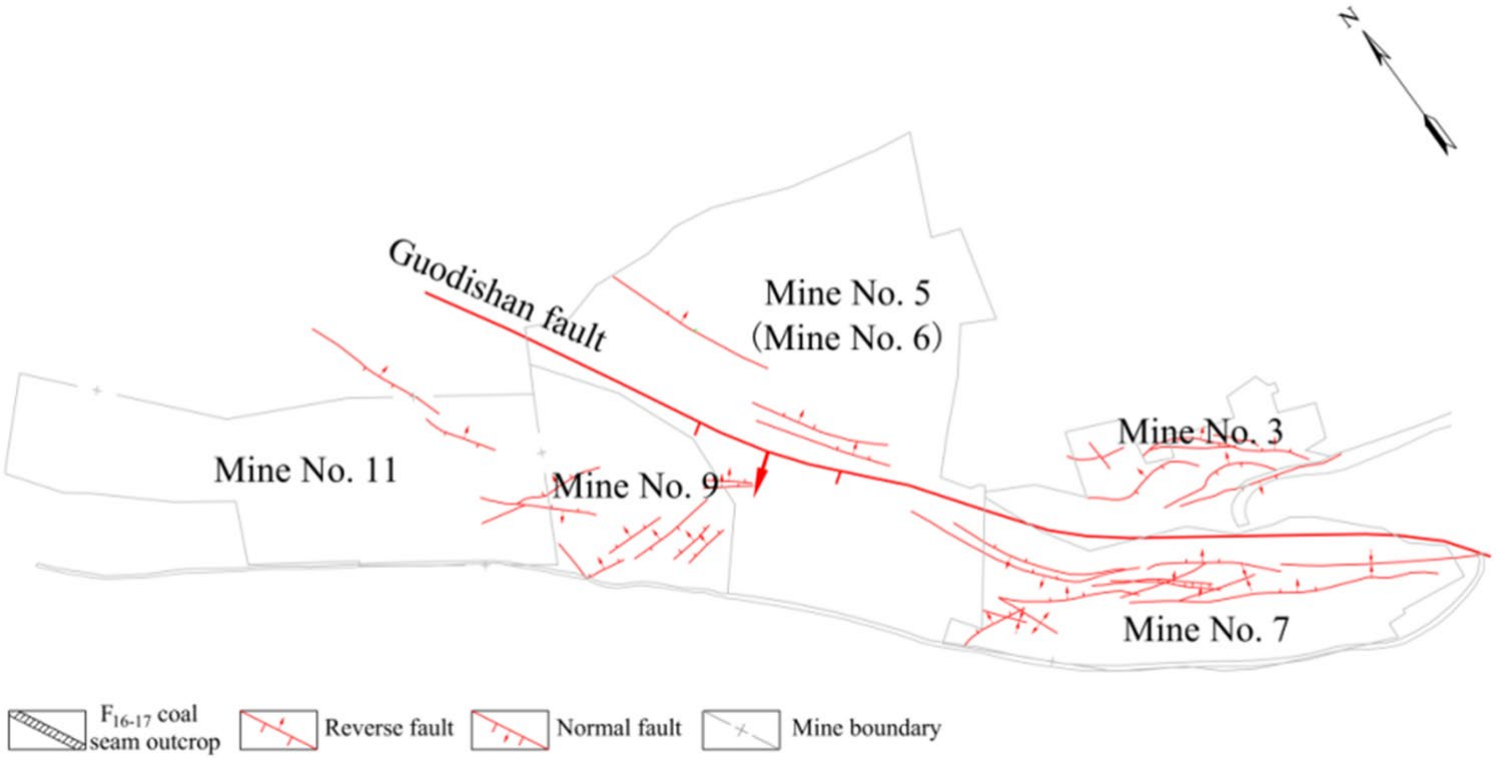
Schematic diagram of main structures in the west of Pingdingshan coal area.

Pingdingshan coal area, located in the central and southern part of Henan Province, is an important large coal producing area in China. Five coal seams in the mining area have been identified as economic resources, among which F_16-17_ coal seam is one of the important mining coal seams due to its excellent coal quality.The Guodishan fault extends 25 km in length and dips 45–75 degrees, which is the main control structure in the central and western regions of Pingdingshan mining area. Its geographical location is shown in Fig. 2.

Guodishan fault has been active since the coal accumulation period, which is a synsedimentary fault^23^. After Indosinian movement and Yanshan movement, the type of fault changed from normal fault to reverse fault. At the early stage of Himalayan movement (E2-E3), the fault underwent dextral tensional and torsional activity, and then transformed into normal fault again. The complex tectonic movement led to the complex geological conditions in the area near the Guodishan fault.

Since the F_16-17_ coal seam near Guodishan fault was mined, coal and gas outburst accidents have occurred repeatedly. In situ stress is one of the main factors leading to coal and gas outburst, so it is necessary to investigate the distribution characteristics of the original geostress near the Guodishan fault.

**Figure 3:**
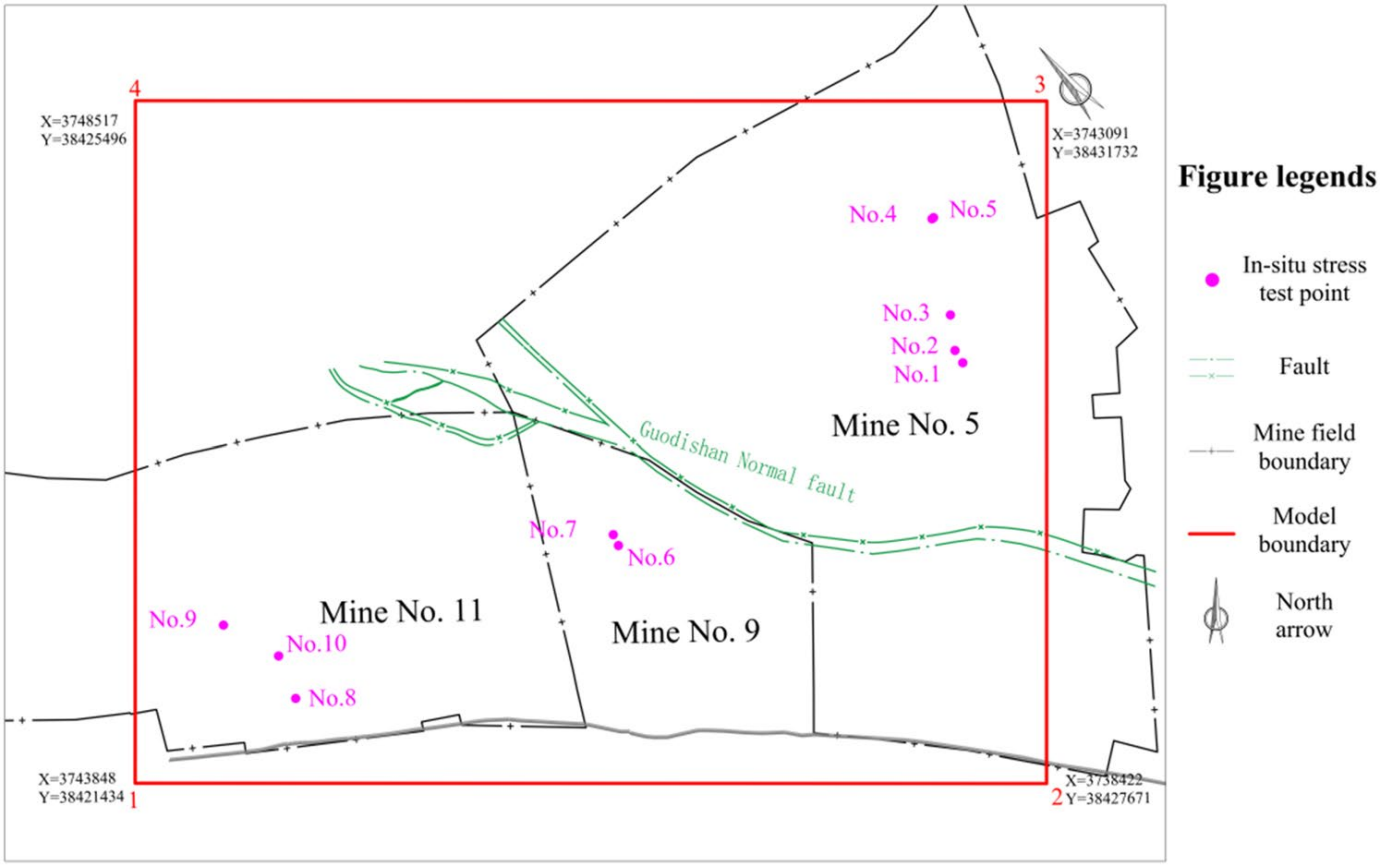
Map of the modeled area.

There are 10 in-situ stresses measurement locations close to Guodishan fault^23^ and an area has been isolated for the study in this paper (Fig. 3).

### 3D geological model development

The geological conditions around the Guodishan Fault is very complicated. There are massive rock layers in the area and massive minor defects originated from the fault. The two sides of the fault undergo a significant displacement and twist due to the tectonic movement, leading to a huge variation in the seam thickness, dip direction and dip angles at the two sides. Such variations lead to a great challenge in geological model development. In this paper, we decided to keep the influential factors to the model as many as possible to make our model more representative to the real scenario. We simplified the geological conditions according to the following.

### Rock types

The coal seams around the Guodishan Fault are located between the Shiqianfeng Formation in Permian Period and Taiyuan Formation in Carboniferous Period. As such, the geological formations that have been selected for modelling is from the bottom of the Taiyuan Formation up to the surface. Referring g to the stratum bar chart of Mine No.5, No.9 and No.11 near the Guodishan Fault, the strata of the geological model are divided according to the age of formation, and the mechanical parameters of each rock layer are referred to the mechanical parameters of the main rocks in the strata, as shown in Table 1. The rock mechanics parameters in the model are derived from previous geological research reports^24^.

**Table 1:** Geological formations.

Rock formation	Rock type	Formation thickness (m)		ρ/kg·m^3^	E/GPa	ν
		Hanging wall	Foot wall			
Cenozoic Erathem quaternary period	Clay	20	20	1970	0.3	0.35
Permian Shiqianfeng formation (Upper)	Medium-fine grained quartz sandstone	130	130	2987	5.8	0.16
Permian Shiqianfeng formation (Pingdingshan)	Medium-coarse grained quartz sandstone	120	120	2640	7.5	0.25
Permian Shihezi formation (A and B)	Mudstone, sandston and coal	310	310	2470	8.7	0.26
Permian Shihezi formation (C)	Mudstone, sandy mudstone and coal	93	93	2490	10.8	0.14
Permian Shihezi formation (D)	Mudstone, siltstone and coal	88	84	2510	9.1	0.19
Permian Shihezi formation (E)	Mudstone, fine sandstone and coal	143	140	2530	8.9	0.25
Permian Shanxi formation (F)	Mudstone, siltstone and coal	97	86	2540	9.2	0.20
Carboniferous Taiyuan formation (G)	Limestone, mudstone, sandy mudstone and coal	60	62	2760	12.1	0.21
Cambrian Gushan formation	Limestone	70	70	2800	14.4	0.2

F_16-17_ coal seam is contained in Permian Shanxi Formation.

### Formation contacts

**Figure 4:**
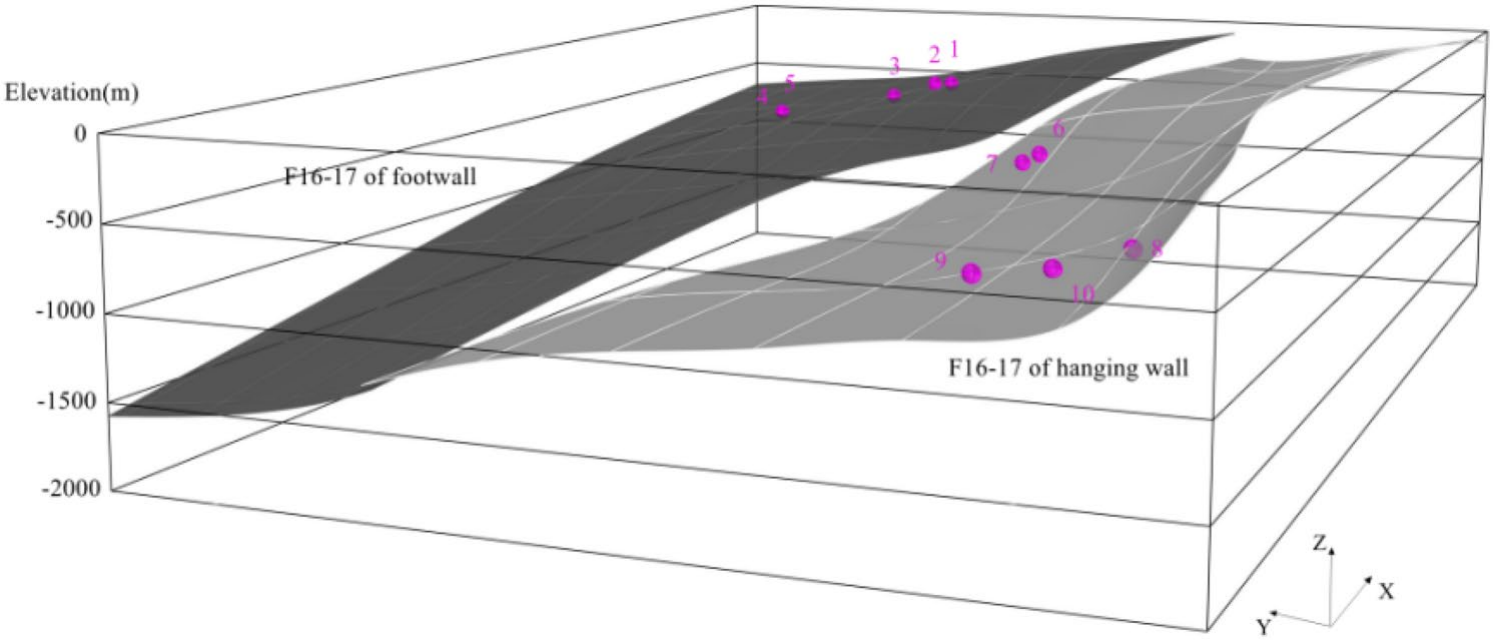
Diagram of coal seam occurrence of F_16-17_. The pink bullets indicate the in-situ stress points (as in Fig. 3).

All formation contacts exhibit significant differences due to the historical tectonic movement and weathering effect. In this study, we determined the distribution of F_16-17_ coal seam through the information of geological drilling, as shown in Fig. 4. We also use remote sensing terrain model data to draw the surface.

### Structures and other factors

The two sides of the faults twisted during the formation leading to different dip angles and dip directions of the hanging wall and footwall. As such, both sides have been modelled separately with hanging wall dipping at 15 degrees towards NE direction whereas foot wall dipping at 10 degrees towards north direction. The minor faults and other structures have insignificant influence on the in-situ stresses and therefore, the major fault is the only one structure that has been modelled.

**Figure 5:**
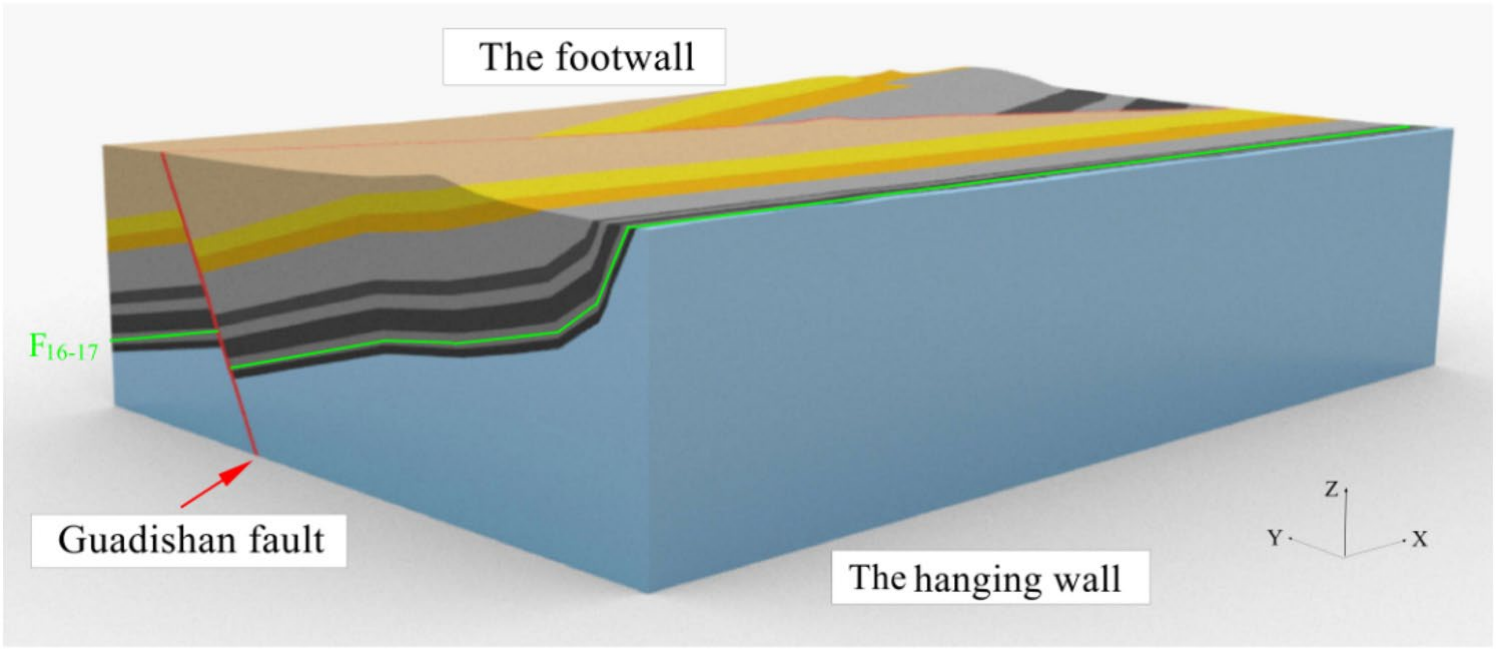
3D geological model of Guodishan fault area.

The geological model is 8.2 km long, 6.2 km wide and 2.05 ~ 2.35 km high as seen in Fig. 5.

Considering the huge scale of the geological model might affect the numerical calculation time and accuracy, we have divided the model into 1,253,871 elements and 82,455 nodes with the help of the built-in mesh generator and Griddle plugin.

## In-situ stresses inversion

### Boundary conditions

The in-situ stress mainly comes from the self-gravity of rock mass and the tectonic force, so this paper only adopts four different stress types: self-gravity of overburden, horizontal compressive stress in the X-axis direction, horizontal compressive stress in the Y-axis direction and X–Y plane shear stress.

Import the model into FLAC^3D^, and set the boundary at both ends of the X axis and Y axis of the model as rolling bearings, the top as free boundary, and the bottom as fixed boundary. Apply self-weight stress, 35 MPa ~ 0 MPa gradient stress in x-axis direction, 18 MPa ~ 00 MPa gradient stress in y-axis direction and 10 MPa stress in xy direction to the model in turn, and conduct simulation calculation.

### Numerical calculation

**Figure 6:**
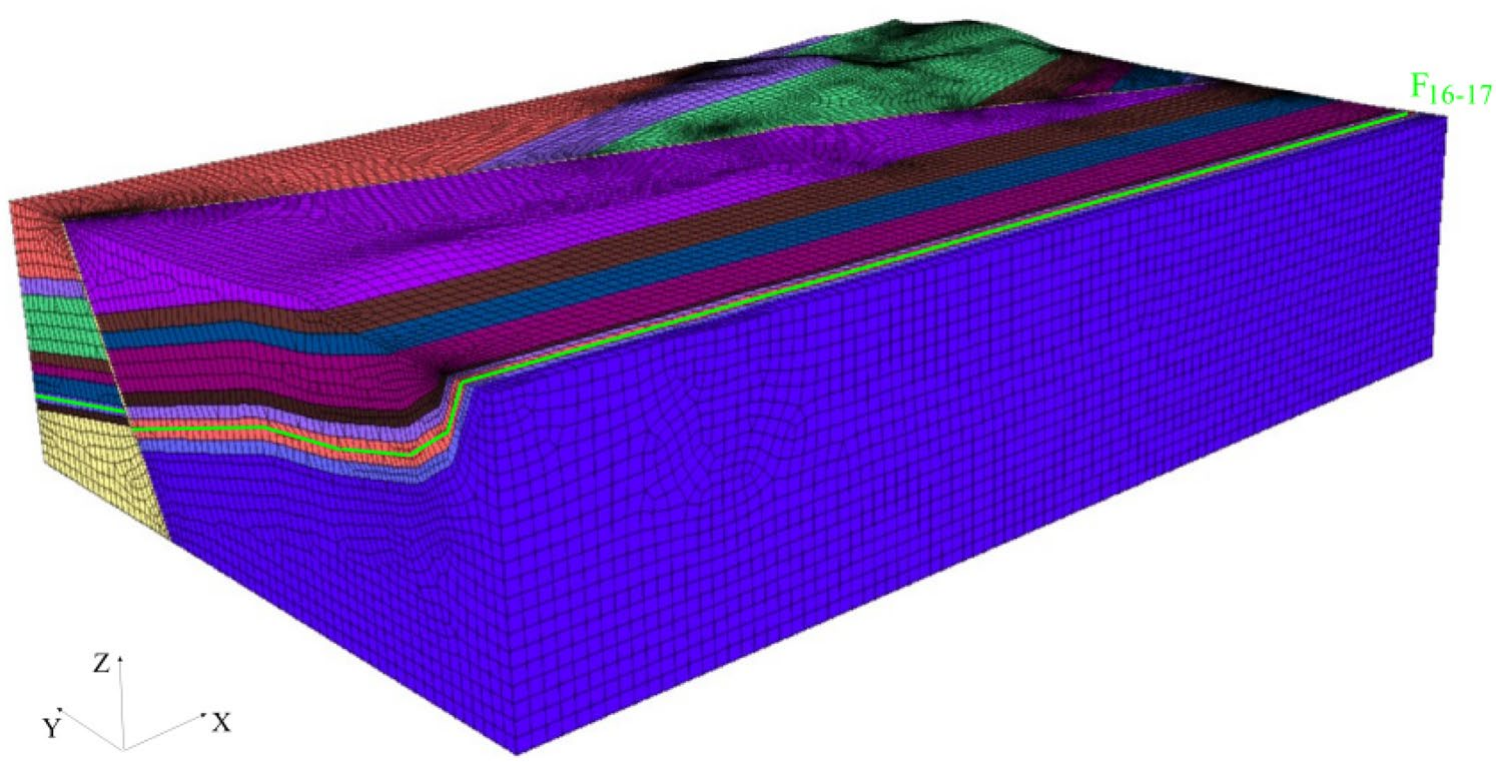
Imported model in FLAC^3D^.

The geological model established in Rhino was implemented into FLAC^3D^ as seen in Fig. 6. A constitutive model based on the isotropic elastomer was set to all the small elements in the simulation. As the strength of the rock in the fault is very low, the input parameters have been adjusted accordingly as seen in Table 1.

Through numerical simulation under four working conditions, the calculated values of in-situ stress can be obtained when four forces are applied respectively. We extract the calculated in-situ stress values at the same location as the actual in-situ stress measuring points. Then the whole model is regarded as a transversely isotropic elastic body, and the horizontal in-situ stress and vertical in-situ stress are regressed by multiple regression fitting respectively. Finally, the regression formulas of horizontal in-situ stress and vertical in-situ stress can be obtained.

**Figure 7:**
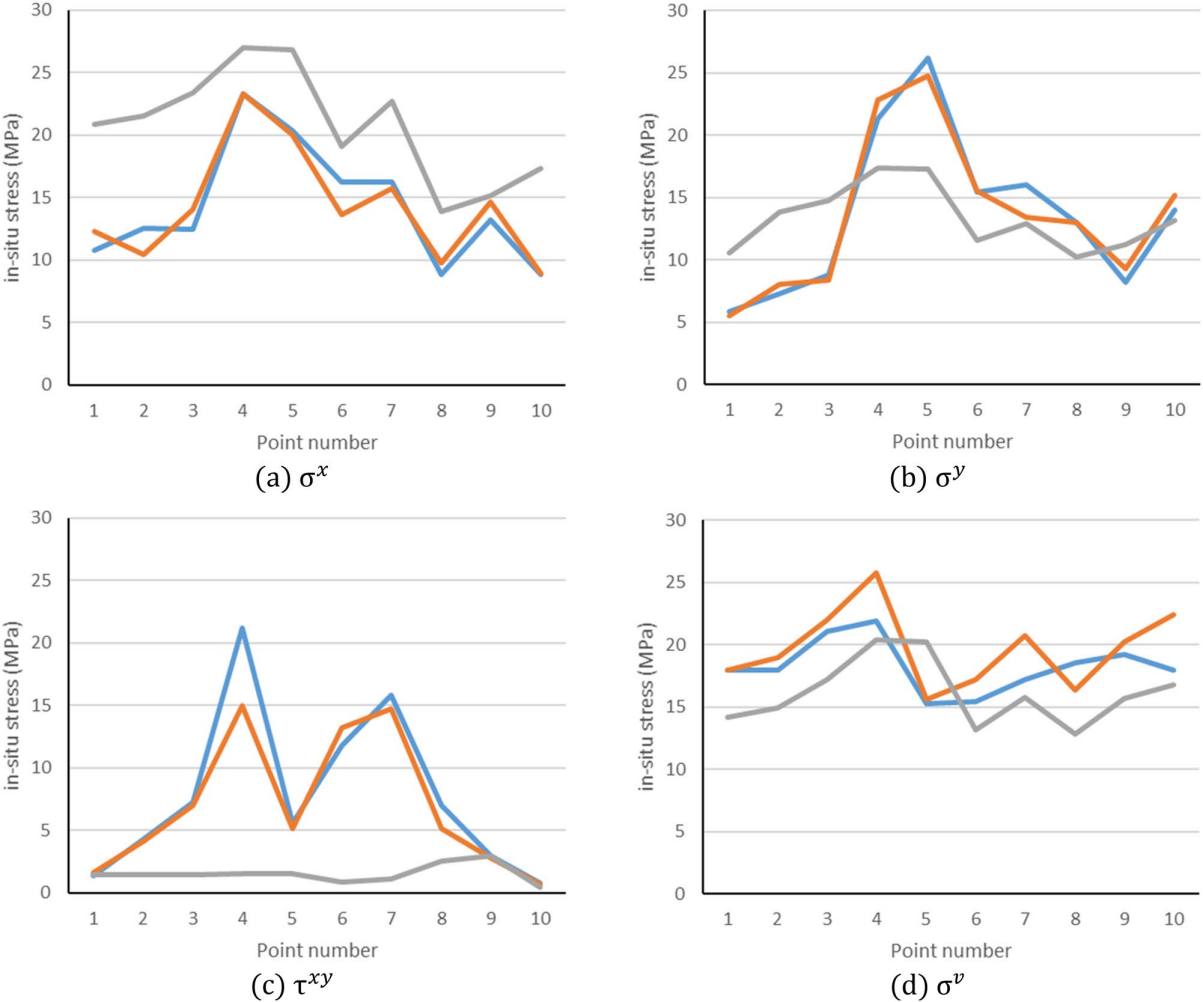
Diagram of comparison between simulated and measured values.

We compared the calculated values of in-situ stress based on the large-scale transverse isotropic model, the calculated values of in-situ stress based on the isotropic model and the measured values of in-situ stress, as shown in Tables 2, 3, and we also drew a comparison chart using Microsoft Office, as shown in Fig. 7.

**Table 2:** Comparison between calculated and measured in-situ stresses based on transverse isotropy.

No.	${\upsigma }^{x}$/MPa			${\upsigma }^{y}$/MPa			${\uptau }^{xy}$/MPa			${\upsigma }^{v}$/MPa		
	A	I	δ/%	A	I	δ/%	A	I	δ/%	A	I	δ/%
1	10.73	12.27	14.35	5.87	5.53	5.79	1.36	1.64	20.59	17.96	17.92	0.22
2	12.51	10.46	16.39	7.25	8.04	10.90	4.27	4.17	2.34	17.96	18.95	5.51
3	12.43	14.05	13.03	8.83	8.34	5.55	7.28	7.03	3.43	21.1	21.99	4.22
4	23.25	23.31	0.26	21.29	22.83	7.23	21.23	14.98	29.44	21.93	25.80	17.65
5	20.33	19.98	1.72	26.17	24.75	5.43	5.58	5.16	7.53	15.27	15.57	1.96
6	16.22	13.59	16.21	15.46	15.49	0.19	11.76	13.22	12.41	15.41	17.23	11.81
7	16.26	15.75	3.14	15.99	13.42	16.07	15.85	14.72	7.13	17.24	20.71	20.13
8	8.88	9.80	10.36	12.98	13.00	0.15	7.03	5.13	27.03	18.57	16.40	11.69
9	13.18	14.63	11.00	8.24	9.33	13.23	2.95	2.76	6.44	19.2	20.19	5.16
10	8.85	8.94	1.02	13.97	15.21	8.88	0.80	0.65	18.75	17.96	22.37	24.55

A, I and δ represent the actual measured value, the inversion value and the relative error.

**Table 3:** Comparison between calculated and measured in-situ stresses based on isotropy.

No	${\upsigma }^{x}$/MPa			${\upsigma }^{y}$/MPa			${\uptau }^{xy}$/MPa			${\upsigma }^{v}$/MPa		
	A	I	δ/%	A	I	δ/%	A	I	δ/%	A	I	δ/%
1	10.73	20.83	94.13	5.87	10.55	79.72	1.36	1.42	4.53	17.96	14.17	21.13
2	12.51	21.54	72.16	7.25	13.82	90.68	4.27	1.44	66.34	17.96	14.91	16.96
3	12.43	23.39	88.18	8.83	14.75	67.08	7.28	1.45	80.11	21.1	17.23	18.34
4	23.25	26.95	15.93	21.29	17.35	18.49	21.23	1.51	92.88	21.93	20.40	6.98
5	20.33	26.82	31.93	26.17	17.27	34.02	5.58	1.52	72.78	15.27	20.21	32.33
6	16.22	19.07	17.57	15.46	11.54	25.33	11.76	0.87	92.62	15.41	13.18	14.45
7	16.26	22.74	39.83	15.99	12.89	19.36	15.85	1.13	92.90	17.24	15.76	8.60
8	8.88	13.84	55.91	12.98	10.19	21.47	7.03	2.52	64.14	18.57	12.86	30.77
9	13.18	15.12	14.75	8.24	11.24	36.41	2.95	2.97	0.53	19.2	15.70	18.25
10	8.85	17.34	95.88	13.97	13.18	5.68	0.80	0.41	48.45	17.96	16.80	6.45

It can be seen from Tables 2, 3 and Fig. 7 that the simulated calculated ground stress is basically consistent with the measured ground stress, which indicates that the three-dimensional geological model is relatively accurate. The relative error of the model based on transverse isotropy is less than 30%, which is more accurate than the isotropic model.

These deviations might be attributed to a few reasons including the ignorance of the small faults in the numerical model leading to a higher discrepancy in the local rock mass, and oversimplification of the numerical modelling and calculation leading to the compromise of the modelling accuracy. Nevertheless, the proposed method based on the transverse isotropic elastomer modelling approach is still accurate enough for the in-situs stresses inversion and can be potentially used for engineering applications with high confidence.

## Inversion results and data analysis

**Figure 8:**
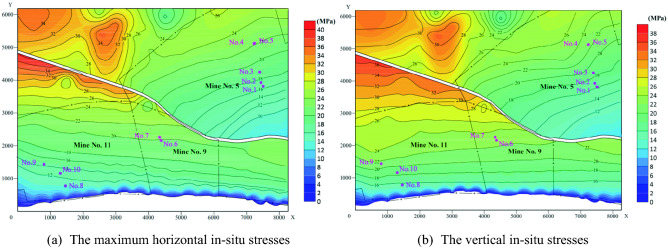
In-situ stresses distribution at the roof of Seam F_16-17_.

In order to study the distribution of ground stress, we selected 280 in-situ stress of F_16-17_ coal seams on both sides of the Guodishan fault, and mapped them into Fig. 8 and Table 4. Figure 8a shows the major in-situ stress distribution along horizontal direction in Seam F_16-17_ and Fig. 8b shows the in-situ stress distribution along vertical direction. It can be seen that as the Seam F_16-17_ dips into the ground, the major horizontal in-situ stress and vertical in-situ stress tend to increase.

**Table 4:** The calculated in-situ stresses at the roof of Seam F_16-17_.

Level/m	Locations	Depth/m	Major horizontal stress/MPa	Vertical stress/MPa	K
− 400	Hanging wall	500	6.4	12.8	0.50
	Foot wall	650	14.4	16.5	0.87
− 600	Hanging wall	700	12.6	17.7	0.71
	Foot wall	850	20.4	21.4	0.95
− 800	Hanging wall	950	20.4	23.9	0.85
	Foot wall	1000	24.9	25.1	0.99

In general, the in-situ stress near the Guodishan fault has the following characteristics:

1. With the extension of F_16-17_ coal seam to the deep, the maximum horizontal principal in-situ stress and vertical in-situ stress have an increasing trend, but the maximum horizontal in-situ stress of coal seams at the same elevation in the hanging wall of the fault is generally less than that of the footwall, indicating that the footwall of the fault is more affected by tectonic stress.
2. The vertical in-situ stress near the fault is generally greater than the maximum horizontal principal in-situ stress, that is, the lateral pressure coefficient K is generally less than 1.
3. With the increase of burial depth, the K ratio of the coal seams on both sides of the fault gradually approaches 1, indicating that the in-situ stress at the depth gradually approaches the hydrostatic pressure state, which is consistent with the findings of previous researchers^25^.
4. When the elevation of the coal seam drops from − 400 m to − 800 m, the maximum horizontal principal in-situ stress in the hanging wall of the fault increases by 2.18 times, compared with 0.73 times in the footwall of the fault. It shows that with the increase of the buried depth of the coal seam, the effect of the tectonic stress is relatively reduced. Moreover, due to the high in-situ stress gradient in the hanging wall, the hazard of hazard induced by in-situ stress in the hanging wall is relatively high.
5. The stress concentration area appears in the contact area between the fault end and the fault wall. In these areas, dynamic disasters caused by high stress may occur, but the farther away from the fault area, the lower the risk of dynamic disasters.

## Conclusions

• Based on the information of F_16-17_ coal seam exposed by geological drilling and actual engineering, this paper has established a more accurate coal seam curved surface and 3D geological model by Rhino software, which explains the advantages of Rhino in building layered and uneven geological model, and also provides a fast and accurate modeling method for in-situ stress inversion.
• The application of large-scale transversely isotropic model in the stage of multiple regression analysis can not only reduce the error caused by assigning the model to isotropic elastic bodies, but also avoid the difficulty in measuring the transversely isotropic parameters of the stratum in the stage of numerical simulation calculation, and improve the accuracy of in-situ stress inversion while reducing workload.
• The inversion results of in-situ stress show that the in-situ stress of F_16-17_ coal seam in the hanging wall of Guodishan fault is greatly different from that in the footwall, and the influence of tectonic stress on the footwall is more effective. And with the extension of the coal seam to the deep, the K ratio gradually approaches 1. It is worth noting that there may be a higher potential risk near the fault, because the stress has changed significantly, which may lead to more serious rock failure.
• The in-situ stress inversion method proposed in this paper, which combines Rhino precise modeling and large-scale transversely isotropic model, has high accuracy and reliability, as can be seen from the results of in-situ stress inversion near Guodishan fault. However, the geological and topographical conditions in the area near the Guodishan fault are complex, so it is unrealistic to accurately simulate the in-situ stress field. The result of in-situ stress inversion is an equivalent and approximate in-situ stress field, which aims to study the in-situ stress distribution near the fault and provide precondition and basis for later research.

## Data Availability

Numerical simulation data sets are available from corresponding authors upon reasonable request.
